# Creating a Culture of Health in Planning and Implementing Innovative Strategies Addressing Non-communicable Chronic Diseases

**DOI:** 10.3389/fsoc.2019.00009

**Published:** 2019-02-26

**Authors:** Chariklia Tziraki-Segal, Vincenzo De Luca, Silvina Santana, Rosa Romano, Giovanni Tramontano, Paola Scattola, Corrado Celata, Giusi Gelmi, Sara Ponce Márquez, Luz Lopez-Samaniego, Veronica Zavagli, Arja Halkoaho, Corrina Grimes, Maria Teresa Tomás, Beatriz Fernandes, Laura Calzà, Patrizia Speranza, Liliana Coppola, Harriët Jager-Wittenaar, Rónán O'Caoimh, Anna-Maija Pietilä, Ana Maria Carriazo, Joao Apostolo, Guido Iaccarino, Giuseppe Liotta, Donatella Tramontano, William Molloy, Maria Triassi, Vincenzo Viggiani, Maddalena Illario

**Affiliations:** ^1^Israel Gerontological Data Center, Hebrew University of Jerusalem, Jerusalem, Israel; ^2^MELABEV- Community Clubs for Elders, Jerusalem, Israel; ^3^Research and Development Unit, Federico II University Hospital, Naples, Italy; ^4^Department of Economics, Management, Industrial Engineering and Tourism, Institute of Electronics and Informatics Engineering of Aveiro, University of Aveiro, Aveiro, Portugal; ^5^Health Protection Agency of the Metropolitan City of Milan, Milan, Italy; ^6^Health Promotion, Screening and Prevention Unit, Milan, Italy; ^7^International Research Projects Office (IRPO), Universidad de Deusto, Bilbao, Spain; ^8^Progress and Health Foundation, Regional Ministry of Health of Andalucía, Seville, Spain; ^9^ANT Italia Foundation, Bologna, Italy; ^10^School of Health Care and Social Services Education and R&D, Tampere University of Applied Sciences, Tampere, Finland; ^11^Public Health Agency of Northern Ireland, Belfast, United Kingdom; ^12^Health and Technology Research Center, Escola Superior de Tecnologia da Saúde de Lisboa, Instituto Politécnico de Lisboa, Lisbon, Portugal; ^13^Department of Pharmacy and Biotechnology, University of Bologna, Bologna, Italy; ^14^General Affairs Unit, Federico II University Hospital, Naples, Italy; ^15^Research Group Healthy Ageing, Allied Health Care and Nursing, Hanze University of Applied Sciences, Groningen, Netherlands; ^16^Department of Medicine, Clinical Sciences Institute, National University of Ireland, Galway, Ireland; ^17^Department of Nursing Science, University of Eastern Finland, Kuopio, Finland; ^18^Regional Ministry of Health of Andalucía, Seville, Spain; ^19^The Health Sciences Research Unit: Nursing, Nursing School of Coimbra, Coimbra, Portugal; ^20^Department of Medicine, Surgery and Dentistry, University of Salerno, Salerno, Italy; ^21^Department of Biomedicine and Prevention, University of Rome “Tor Vergata”, Rome, Italy; ^22^Department of Molecular Medicine and Medical Biotechnology, Federico II University of Naples, Naples, Italy; ^23^Clinical Gerontology and Rehabilitation Centre, Gerontology and Rehabilitation School of Medicine, University College of Cork, Cork, Ireland; ^24^Department of Public Health, Federico II University of Naples, Naples, Italy; ^25^Director General, Federico II University Hospital, Naples, Italy; ^26^Health Innovation Division, General Directorate for Health, Naples, Italy

**Keywords:** culture of health, active and healthy aging, inclusive health care, salutogenesis, health innovation

## Abstract

Ongoing demographic changes are challenging health systems worldwide especially in relation to increasing longevity and the resultant rise of non-communicable diseases (NCDs). To meet these challenges, a paradigm shift to a more proactive approach to health promotion, and maintenance is needed. This new paradigm focuses on creating and implementing an ecological model of Culture of Health. The conceptualization of the Culture of Health is defined as one where good health and well-being flourish across geographic, demographic, and social sectors; fostering healthy equitable communities where citizens have the opportunity to make choices and be co-producers of healthy lifestyles. Based on Antonovsky's Salutogenesis model which asserts that the experience of health moves along a continuum across the lifespan, we will identify the key drivers for achieving a Culture of Health. These include mindset/expectations, sense of community, and civic engagement. The present article discusses these drivers and identifies areas where policy and research actions are needed to advance positive change on population health and well-being. We highlight empirical evidence of drivers within the EU guided by the activities within the thematic Action Groups of the European Innovation Partnership on Active and Healthy Aging (EIP on AHA), focusing on Lifespan Health Promotion and Prevention of Age-Related Frailty and Disease (A3 Action Group). We will specifically focus on the effect of Culture on Health, highlighting cross-cutting drivers across domains such as innovations at the individual and community level, and in synergies with business, policy, and research entities. We will present examples of drivers for creating a Culture of Health, the barriers, the remaining gaps, and areas of future research to achieve an inclusive and sustainable asset-based community.

## Background

We are currently facing exceptional demographic changes, as longevity increases while falling fertility rates create labor shortages. New waves of urbanization and the rise of NCDs in aging populations have been eliciting complex challenges in social and health systems worldwide, particularly in western countries. Although urbanization is generally associated with improvements in income levels and health outcomes, pressure from these demographic changes is inducing social and health inequalities in cities and in rural areas that undergo a depopulation phenomenon. Worldwide, non-communicable chronic diseases (NCCD) are on the rise because of unhealthy urban lifestyles and inadequate service provisions. According to the World Health Organization (WHO), 86% of the deaths and 77% of the loss of healthy life years in Europe are caused by chronic diseases (cardiovascular disease, cancer, diabetes mellitus, chronic respiratory disease, mental health problems, and skeletal muscle disorders). Approximately €700 billion are spent every year on the treatment of chronic diseases across the EU which has the highest burden of NCCD worldwide. Cardiovascular disease and cancer cause nearly three-quarters of mortality in the region, where three major groups of diseases—cardiovascular disease, cancer, and mental disorders including cognitive decline/dementia. An impressive percentage of health care resources are spent on their treatment. To face these challenges, a new culture of health is required, which takes into account disease prevention and health promotion activities aimed at strengthening individual, environmental and social resources. Such improved well-being integrates mental health and physical health and results in holistic approaches to disease prevention and health promotion across the lifespan. Individuals with high levels of well-being are more productive at work and more likely to contribute to their communities. In this context, measures of subjective well-being are key political issues compared to Gross Domestic Product (GDP), since subjective well-being better captures the quality of life of a nation's citizens, and lead to policies that are more effective and equitable. In this new paradigm, health crosses the road of well-being beyond the traditional boundaries of health care delivery systems. Existing health care systems are fragmented, reactive, and costly. Attempts to change this model started in 1998, when “Improving Chronic Illness Care” created the Chronic Care Model (CCM) (Alleyne et al., 2010; Gee et al., 2015; World Health Organization, 2018). The CCM identifies the essential elements of a health care system that encourages high-quality chronic disease care: the community, an integrated health system, empowerment and self-management of active and informed patients and providers, delivery system design, decision support, and clinical information systems. The CCM, however, does not adequately addresses the proactive approach toward well-being even in persons who have NCD (Diez Roux, 2001; Krieger, 2001; McMichael, 2002; Trujillo and Plow, 2016; Acosta et al., 2018). Well-being can be referred to the complex array of physical, psychological, social, economic, geographical, cultural factors that exert a powerful influence on our lives and our health, not just “absence of illness.” In exploring a more ecological model of addressing well-being across the lifespan even in the presence of NCD, we will explore the general principles and evolution of the Salutogenesis as it applies to well-being. Antonovsky's Salutogenesis theory assumes that the ecosystem works as a whole since its focus is on creating a “new higher state of health than the one is currently experienced” by the individual or the system (Antonovsky, 1979, 1996). The Salutogenesis theory can be applied at a societal level, but also at the individual and a group level. The key concept of the theory is the sense of coherence (SOC) which means the ability to comprehend the whole situation and the capacity to use available resources. This capacity is a combination of peoples'/organizations/ability to assess and understand the situation they are, to find a meaning to move in a health promoting direction, as well as the capacity to act. The three dimensions of sense of coherence are: comprehensibility, meaningfulness, and manageability (Lindström and Eriksson, 2005).

**Figure 1:**
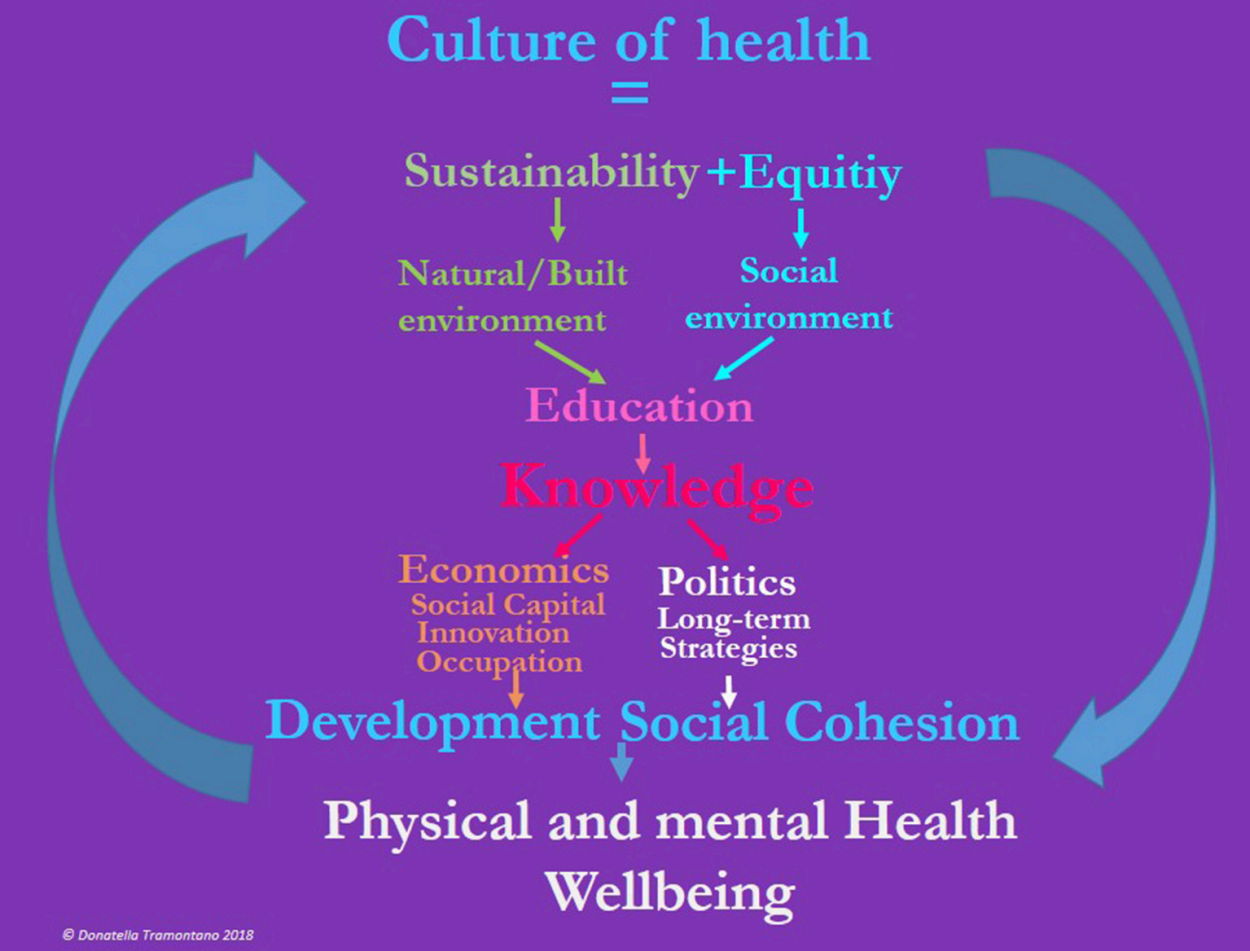
The determinants of health according to the CSDH.

It is becoming increasingly clear that to improve population health, it is necessary to engage all sectors toward well-being, equity, and multiple domains as depicted in Figure 1. These synergies are not simply linear (cause-effect) but rather impact and are impacted along multidirectional domains and are essential to creating a Culture of Health based on the concepts of the Social Cohesiveness Salutogenesis theory, as depicted in Table 1. Although. the Salutogenesis theory with its multidimensional components has yet to be applied at a large scale and across various entities, there are signs that this is happening. A growing number of communities, regions, and states are redefining what it means to get and stay healthy by addressing the multiple determinants of health, among diverse sets of stakeholders. These developments in health and society present a chance to catalyze a grassroots cross-border movement demanding and supporting a widely shared, multifaceted vision for a Culture of Health (Huber et al., 2011; Lavizzo-Mourey, 2017). One's notion about what it means to be healthy is influenced by their own culture (Maoz et al., 1977, 1978), therefore making health a shared value is central to building a culture of health. In this article, we define a new action framework—a culture of health, as a conceptually important construct to spur faster progress toward equitable health outcomes across borders and regions (Chandra et al., 2016).

**Table 1:** The three dimensions of sense of coherence according to the Social Cohesiveness Salutogenesis theory.

**Key concepts of sense of coherence (SOC)**	**Content**
Comprehensibility	The extent to which person/entity perceive the stimuli that confront them, deriving from the internal and external environments, as making cognitive sense as information that is ordered, consistent, structured, and clear. It is the way of perceiving and understanding the world and own place in it. e.g., health perceptions, body image, self-health, comprehension, therapeutic patient education. e.g., health perceptions, body image, self-health, comprehension, therapeutic patient education. The cognitive component of the SOC. The cognitive component of the SOC.
Meaningfulness	Refers to the extent to which a person feels that life makes sense emotionally, that problems and demands are worth investing energy in, are worthy of commitment and engagement, seen as challenges rather than burdens. Relates to the emotional side of the overall attitude to life and its events, e.g., instrumental value of health, the absolute value of health, counseling and support. The emotional component of the SOC.
Manageability	The extent to which an individual entity assesses the resources and abilities readily available to meet the needs, e.g., external sources such as professional support, family coherence, work and leisure time, social support, self-management skills, and minimizing the discomfort of change. Internal support: self-imagine, self-help, attitude. The instrumental/behavioral component of the SOC.

Our aim is to create a proactive agenda for immediate action toward creating a “culture of health” as part of a shared cultural value across the European Union (EU), as expressed in Lisbon Treaty, in the EU Health Strategy and the European Innovation Partnership on Active and Healthy Aging (EIP on AHA) (Lagiewka, 2012; García, 2013; Bousquet et al., 2015, 2017; Malva and Bousquet, 2016; Illario et al., 2017; Liotta et al., 2018a; Malva et al., 2018).

The study led by the Commission on Social Determinants of Health (CSDH) set up by WHO summarizes the evidence on how the structure of societies, through multiple social interactions, norms, and institutions, influences population health and provides an overview of what governments and public health can achieve (WHO, 2010a). The CSDH framework departs from many previous models by conceptualizing the health system itself as a social determinant of health (SDH), especially in reference to service accessibility and sustainability, to differences in exposure and vulnerability, and through inter-sectoral actions led from within the health sector (Figure 1).

The building blocks for creating a culture of health include, first and foremost, health education at all levels of society, training programs, and policies that support and encourage person-centered care of well-informed citizens and community/policy leaders. Large-scale adoption of digital solutions can support prevention, healthy lifestyles, and integrated care as the new drivers to improve the quality of life throughout the lifespan (CSDH, 2008).

The determinants of health and SDH, such as biological, physical, behavioral, environmental, and social factors, are associated with the individual perception of illness or health. Yet, the health level and perceived quality of life of individuals are determined by several factors, policies, and conditions that lay mainly outside the control of individual concerns. Therefore, efforts to improve population health should be centered not only in the health care system but also on the conditions where individuals are born, live, work, and age (Lock, 2000).

The determinants of health especially referred to social factors can be associated with health inequalities, that have been targeted by national and international efforts (Goldblatt et al., 2015). Chandra et al. (2016) propose 3 drivers of health (e.g., priorities or focus areas) to develop new shared values to drive health promotion and improve individual and population health. The first one is mindset and expectations, that is related to the way individuals and communities view health and well-being, and to their responsibility to advocate for improvements in policies, environments, and services promoting health. The second driver is the sense of community that fosters identity of individuals based on feelings of membership, belonging and shared experiences. The last driver is civic engagement, a process combining individual knowledge, skills, values, and motivation to make a difference in the civic life of the community.

In the same perspective and aiming to improve health equity through action across the life course, the European “DRIVERS for Health Equity” project (Grant number #278350); project was created, establishing recommendations to improve health inequalities at 3 actions areas (Goldblatt et al., 2015). The first one is early child development because adversity at this stage of life has profound effects and outcomes on cognitive domain affecting communication and language, social and emotional skills. The second driver is fair employment, as employment and working conditions impact directly and indirectly on the health of individuals. The third driver is income and social protection, as income and living conditions influence an individual's health and variations between social groups. Social protection can mitigate the consequences of income loss.

Drivers and shared values influence health policies through decisions, plans and/or actions that are undertaken to achieve specific health care goals in society. Drivers also set strategies that proportionate universalism in health, through the prioritization of investments, sharing responsibilities, and providing equitable health opportunities.

Efforts to improve population health traditionally center on the healthcare system as the key driver, orienting the search for drivers of health toward the healthcare systems, despite the evidence that the effects of medical care are limited in determining who becomes sick or injured. Accordingly, medical care accounts for 10%, similarly to biology and genetics, while social factors and healthy behaviors account for 70%. Thus, improving population health requires broader approaches that address social, economic, and environmental factors, since to cure is the responsibility of the healthcare system, whereas to care is a responsibility of the whole society. Mounting evidence supports causal relationships between many social—including socioeconomic—factors and health outcomes, not only through direct relationships but also through more complex pathways often involving bio-psycho-social processes. In modern society, we undergo a daily array of low-level chronic stress, and our body is continuously in the stress response mode causing insufficient recovery, recognized as an increasing public health concern because of its long-term effects on health and on NCD (Sluiter et al., 2000; Nilsson et al., 2011; McEwen). Social stressors jeopardize the health, quality of life and overall well-being, lowering physical, and mental well-being. The molecular mechanisms relating stress and health are being clearly identified: chronic stress impairs the immune system, increases the production of molecular mediators of stress such as free oxygen radicals, and induces a chronic level of inflammation, which in turn is a key factor in the onset, progression, and outcomes of most common NCD. Tawakol et al. (2017) state that psychosocial stress resulting from adversity is a precipitant of morbidity, as it is associated with an increased risk of cardiovascular disease.

An unequal social context harms health directly, also driving individuals into detrimental coping mechanisms and behaviors, such as drug and alcohol abuse, compulsive eating, gambling, and violence. Moreover, inequality harms health indirectly eroding societal trust and destabilizing communities, endangering social cohesion. Emerging drivers of a new “Culture of health” are represented by equity, social cohesion, solidarity, social justice, and sustainability. Figure 1, dramatically depicts the rather complex and multidirectional impact of the social/biological/environmental domains that can produce a “Culture of Health” model for the whole lifespan and across cultural and geographic contexts.

## Defining the *Culture of Health* in the Framework of Societal Challenges

The WHO in 1946 defined health as a state of physical, mental, and social well-being and not simply the absence of disease or infirmity (WHO, 1946), viewing health holistically. During the first international health promotion conference in Ottawa in 1986 (WHO, 1986) health started to be seen as a process enabling people to develop through their assets, achieving well-being despite the presence of disease.

This new concept of health and well-being contrasts the pathogenesis model, which has a biomedical focus, missing the holistic goals of health and well-being in the prevention and care of chronic and lifestyle-related diseases (Povlsen and Borup, 2015). Opposite to pathogenesis, the Salutogenesis model (Antonovsky, 1979) focuses on factors that promote, increase and maintain well-being (Antonovsky, 1996). Salutogenesis does not view health only as a biological asset, rather as a psychosocial concept and a resource. This means that not just body, mind, and the close environment but also society, and how the individual manages to act and live in it, influence health (Olivius et al., 2004).

Health becomes a positive concept emphasizing social and personal resources, as well as physical capacities. According to the Ottawa Charter, improvements in health need three basic prerequisites: advocacy for health, enabling (taking action in partnership with individuals or groups to empower them) and mediation (different interests of individuals and communities, and different sectors are reconciled to promote and protect health). Health promotion is concerned with action and advocacy to address the full range of potentially modifiable determinants of health—not only those related to the actions of individuals, such as health behaviors and lifestyles, but also factors such as income and social status, education, employment and working conditions, access to appropriate health services, and the physical environments. Achieving change in lifestyles and living conditions that influence health status represents intermediate health outcomes (WHO, 1998).

In this framework, it is relevant considering the changing patterns of diseases, where infectious diseases as the major cause of morbidity and mortality have been replaced by diseases related to individual lifestyle and environmental factors, by cancer, mental diseases and autoimmune and metabolic disorders (Povlsen and Borup, 2015). Some authors referred to this phenomenon as “waves” of diseases and referred to NCDs as “civilization diseases” (Hjort, 1993). In 2008, according to the WHO (Malva et al., 2018), NCDs accounted for two-thirds of global deaths. These diseases are costly for society and the economy, hence the importance of reorganizing the healthcare system to improve their management (Povlsen and Borup, 2015), taking into account knowledge and practical skills, as well as the psychological and social support required to enable individuals and family to adapt and acknowledge the disease (Povlsen and Borup, 2015).

Investing in prevention and control of NCDs reduces premature deaths, preventable morbidity, and disability. At least 86% of deaths and 77% of the disease burden in the WHO European Region are caused by this large group of disorders that have in common determinants (social, economic, etc.), modifiable risk factors and prevention strategies (Figure 1). Action must be directed not only at the individual but also at the social and living conditions, that interact to produce and maintain these behavioral patterns. There is no “optimal” lifestyle to be prescribed for all people: culture, income, family structure, age, physical ability, home, and work environment make certain ways and conditions of living more attractive, feasible and appropriate (WHO, 1998). All these variables have been extensively studied and reviewed in the newly published Salutogenesis handbook (Pelikan, 2017).

## Evidence-Based Emerging Domains for Building a *Culture of Health*

### Multilevel and Multidomain Well-being Assessment

Well-being is a comprehensive concept including individual health, as objective status and subjective perception. Quality of life (QoL) is the more appropriate approach to measure individuals' well-being because of its capacity to capture both the individual expectations and the objective health status. These questionnaires are mainly used to compare the QoL of individuals before and after an event or an intervention more than to evaluate the population health status, due to the subjective component of the assessment (Lins and Carvalho, 2016). The health status of a population is difficult to measure because it is hard to define among individuals, populations, cultures, or even across periods. The Healthy Life Years (HLY) expectancy is an indicator attempting to estimate the health status of the population in a country and is related to factors that also include prevention programs. The measure of HLY expectancy is based on self-reported data, affected by respondents' subjective perception as well as by their social and cultural background (European Union, 2018). However, it can be considered an indicator of the prevention programs impact on the population, and of the trend of this impact during the years. Life Expectancy (LE) can also be considered an objective indicator of the population health status, influenced by prevention programs as well as by clinical activities, reflecting the change of mortality at all ages over the years. The combination of HLY and LE is an effective way of depicting population health status, comparable to Infant Mortality Rate as an indicator of social and economic condition at the country level. However, the need for more specific information at the population level to plan health and social care is emerging (Rijken et al., 2017). The increasing prevalence of chronic diseases pushes the carers community (professionals and informal caregivers) to move the aim of their interventions from pursuing a cure to taking care, including patient-relevant outcomes such as frequency of hospital admission or institutionalization and clinical outcomes (Rijken et al., 2017). Bio-psycho-social frailty is a multidimensional measurement of the risk implied by worsening of quality of life: it is characterized by a loss of physiological reserve, often in the setting of limited socioeconomic resources that results in increased vulnerability to adverse healthcare outcomes (Liotta et al., 2016b). Bio-psycho-social frailty is a comprehensive assessment of the risk of functional decline affected by social and economic domains as well as by functional status and psycho-physical impairment. Many evaluation tools of frailty at community level take the hospital admission, institutionalization, and mortality as indicators of frailty as well as of health and social care service performance (Gilardi et al., 2018). The combination of individual assessment of frailty with big data information stemming from standardized data flow could represent in the future the appropriate approach to assess well-being at both population and individual level and plan effective social and health care services.

### Community Based Synergies for a Sustainable Healthy, Active Lifestyle, and Social Connectivity: The Challenges of an Aging Population

#### The Case for Older Adults

The heat wave that hit Southern Europe during the summer of 2003 caused a relative increase of unexpected deaths in older adults (García-Herrera et al., 2010) especially among the +75 individuals living alone, revealing the deadly impact of the combination between social isolation and psycho-physical impairment. Social isolation is a well-known risk factor for mortality, with maximum impact among older adults, where it is more important than smoking (Holt-Lunstad et al., 2015). However, a systematic preventive approach aimed at reducing social isolation is not pursued by the health systems, although a program aimed at identifying isolated and/or sick individuals, supporting social interventions, might show the same protective impact as a “natural” network of relationships.

Social connections at the population level are weakening, where the most popular living arrangements are living alone, and older age is associated with higher risk of unexpected adverse events. The percentage of people declaring they cannot count on someone in case need is about 19% in Italy, close to 28% among the +75 individuals. Heat-related mortality, as an extreme climate event, hitting mainly the frail part of the population tests the resilience capacity at the population level, related to the Salutogenesis theory and the multidomain cross factors as depicted in Figure 1. However, a similar impact of the heat-waves occurred in Italy in 2003 and 2015, when older adults' mortality showed the limits of the preventive action in the field (Cho et al., 2017).

The experience of “*Viva gli Anziani!*” (Long live older adults) program (Comunità di Sant'Egidio, 2010), running in several Italian cities for 14 years by the Community of Sant'Egidio, shows the potential impact of a social program aimed at protecting socially isolated individuals and increasing the social capital at the community level. The “*Viva gli Anziani!*” program promotes a proactive approach to reach the whole targeted population. According to the risk of a negative event, as assessed by the multidimensional evaluation of frailty offered to all participants, an individual care plan is drafted, and the client is included on the list for periodical phone calls: the higher the risk of adverse events, the more frequently the person will be called, with a maximum frequency of once every 2 weeks. The activities of the program are intensified when a heat wave occurs: individuals +75 are traced by phone, and if necessary, the staff intervenes with a home visit, bringing food and/or medicines as necessary, or involving the client's network of relationships. Over the years, the operators act as a liaison between older adults assisted by the program and the community, in order to increase the social capital of both. The impact of such a program is the limitation of the mortality increase during heat waves (in 2015 in Rome the mortality rate increase was halved among the participants compared with the non-participants who lived in the adjacent urban areas) with 10% annual reduction of hospitalization rate and halving of the annual institutionalization rate (ISTAT, 2018; Liotta et al., 2018b).

#### Case Studies of Salutogenesis Model in NCD

A series of papers from Finland addressed the health promotion and management of metabolic syndrome and type 2 diabetes, utilizing the Salutogenesis model (Halkoaho et al., 2014; Miettola and Vilanen, 2014; Voseckova et al., 2017). In the case of diabetes, counseling was highly traditional, including nutrition, exercise, and medication. The counseling was disease-centered and focused on medication instead of individual everyday life or health-promoting and empowering aspects, such as meaningfulness and manageability. The study showed that health-promoting recourses are not easy to recognize or quantify. Therefore, more teamwork between different stakeholders is needed. In practice, responding to the patients' needs, and especially with regard to health-promoting recourses in counseling, requires more education.

In addition to diabetes, cancer is now the second most common NCD and the WHO recommended approaching patients and their caregivers as a “unit of care,” focusing on the overall well-being of the patient-caregiver dyad rather than just on the patient. An approach most easily addressed within the Salutogenesis theory and the complex multidimensional aspects of well-being as shown in Figure 1.

Family caregivers are the supporting column of any long-term care system and are essential health team members: they play a key role in the management of patients with cancer and provide caregiving activities once provided only by professionals. Often, they are not adequately trained or prepared, and it is well-known that caregiving to a family member with cancer has health implications. Those caring for individuals with chronic diseases are more likely to experience insufficient time for sleep, self-care, and exercise and to face social isolation (Grov et al., 2006; Robison et al., 2009). Caregivers show high levels of stress, depression, greater use of prescription drugs and alcohol use and show a higher mortality rate (Zavagli et al., 2012, 2016). Therefore, supporting informal caregivers effectively is beneficial for the patient-carer dyad and public finances.

The impact of caregiving on caregivers' life depends more on personal psychological resources than on objective caregiving demands or social resources. According to the Salutogenesis Model, the Sense of Coherence (SOC) has a key role in this process. SOC is similar to a coping disposition (Winger et al., 2016) and reflects a person's view of life and capacity to respond to stressful situations, a global orientation to see the world as comprehensible, manageable and meaningful.

Thus, it becomes important to integrate caregivers into formal healthcare settings, that *Associazione Nazionale Tumori* (ANT) Foundation does in Italy at a community level. ANT is an Italian non-profit organization providing critical support in home settings to the patient-caregiver dyad throughout the cancer trajectory, from diagnosis through survivorship, palliative care or bereavement. It does this both from a clinical and research viewpoint (Casadio et al., 2010). Health promotion efforts benefit from strengthening SOC (Super et al., 2015), as learning to cope effectively and developing resilience is beneficial in oncology, and palliative care and people may be “trained” to resilience. Patient and family empowerment is important to strengthen existing general resistance resources (GRRs), create new ones and make them available for people to be aware of, identify and benefit from them. Only in this way, empowerment strategies can increase patients' abilities to manage their disease, adopt healthier behaviors, and use health services more effectively, while also increasing the coping skills and efficacy of their caregivers.

This Salutogenic concept is applicable at different levels: interventions and treatment of groups and individuals (e.g., meaning-centered interventions, mindfulness-based stress reduction) and public health policies (societal level).

### Co-development of Technological Innovations With End-Users

Despite the advancement of technological solutions to sustain independence and well-being of the aging population, the number of the innovations moving from the research field to the clinical scenario or to the market is a single digit fraction. There are many reasons for this peculiarity, and some are summarized below.

(1) The idea behind a technological solution is immature or not appropriately developed. This is the case that occurs when the reasoning behind technological advancement, although academically sound, does not correspond to a true issue for the user foreseen for that technology. This situation might occur when the analysis of the end-user needs is not appropriately carried out.

(2) The technological solution, although appropriate, is not user-friendly, and therefore difficult to be picked up. This scenario might occur when the final end-user is not skilled enough to use that technology, or that technology implies a long learning curve that cannot be completed.

(3) The technology is appealing, the end user picks it up fast and then loses interest, returning to their usual behavior, dismissing the technology. This phenomenon, which is referred to as the “Pokémon-Go Effect” (Wong et al., 2017; Visco et al., 2018), occurs when the real usefulness of the technology is not very well-understood by the end-user, does not change her/his daily life and therefore it is no longer used.

The underlying common feature in the failure of technology innovation uptake is the lack of end-user participation in the development of the technology itself. This issue is so impelling that the approach of the European Commission for digital health innovation suggest to set up a “System for Change,” establishing multi-stakeholders collaboration (including end-users), identifying the real needs the innovations will address and building a strategy on that basis (European Commission, 2014).

The construction of the cultural ecosystem for innovation of health and care should be based on multidisciplinary and multi-actor collaborations. “Living labs” are active laboratories where it is possible to assess “creative” ways to improve health and well-being in the local context while facilitating implementation of innovations and the use of good practices and experiences gained at local, national and international level. The logic of these ecosystems is “user-driven,” and focuses on the involvement of users of services in the planning, experimentation, and implementation of innovative approaches aimed at improving health and well-being, with an “iterative” modality, to scale-up on the basis of experience. This approach stimulates collaboration among the stakeholders of the healthcare innovation process, such as patients, professionals, researchers, social service providers, education system, industry (Kujala, 2003; Bodker et al., 2004; Niitamo et al., 2006; Omachonu and Einspruch, 2010; van Velsen et al., 2015; Vollenbroek-Hutten et al., 2015; Liotta et al., 2016a).

### Creating Synergies Among all Stakeholders to Strengthen Well-being in the Workplace: The Contribution of Business/Companies and Health Care Facilities

Work, health and community are related. Work influences employees mental and physical health (Burton, 2010). On the other hand, the physical and the mental health of workers affects the enterprise: when sick, employees' productivity at work decreases, medical cost as well as absenteeism and presenteeism related cost increase, the quality of work and overall participation become compromised (Cockburn et al., 1999; Goetzel et al., 2004; World Health Organization Regional Office for Europe, 2005; Ulrich et al., 2016). In the sixth European Work Conditions Survey (Eurofound, 2017) almost one in every five workers in the EU28 (18%) reported an illness or health problem lasting more than 6 months. More than half (54%) of those reporting chronic disease also stated that their daily activities are limited because of their health problem, and only 21% said that their workplace or work activity changed to accommodate their health condition.

As workplaces exist in communities and societies, they also have a significant impact on workers' health and enterprise outputs. Today organizations recognize the vital role they play in the development and well-being of society, and that their duties go beyond their financial obligations and legal requirements (Krainz, 2015; Litchfield et al., 2015). They assume social, ethical, and environmental commitments that incorporate in their daily practice, to meet stakeholders' expectations and boost their competitiveness. The organizations committed to corporate social responsibility (CSR) significantly impact their employees' health and well-being (Krainz, 2015).

In the actual context of an aging workforce with NCD, there is a need maintain or improve workers' physical, mental and social well-being and ensure high levels of work engagement, by addressing a number of factors at individual, job and team, organizational but also non-work and societal levels (Yaldiz et al., 2017; Zacher et al., 2018) and by implementing effective measures in this regards, such as work design, health, and performance management and transitions to retirement and bridge employment are particularly important (Zacher et al., 2018).

Health, cognitive abilities, and work motives change with age. Therefore, workplaces need to be designed and adapted to account for age-related changes in physical and mental abilities, maintain and uphold workers' well-being and prevent health challenges and disabilities (Zacher et al., 2018). Flexible working arrangements may benefit the physical and mental well-being of older workers as they allow workers to disengage from stressful activities and develop an identity outside work that can help ease their transition to retirement (Zacher et al., 2018). Workers suffering from an NCD will find it easier and less challenging to accommodate medical treatments, rehabilitation sessions, and episodes of tiredness and weakness related to their illness. Moreover, worksites are important places for promoting health among adults, as a great part of the adult population of a country is usually in the labor market and many employed people spend a significant part of their time at work (Riekert et al., 2014).

The discussion so far stresses the need for complex, multilevel approaches involving a variety of stakeholders from within the companies and the society. In Europe, a number of government and social partners measures aimed at keeping older workers in the labor market have been identified (Eurofound, 2013), including: comprehensive initiatives (national strategies and programs as well as social partner agreements), employment and skills developments, health and work environment improvement, working organization, working time and changing attitudes. Examples addressing health and work environment include: compulsory bargaining on health and safety for companies with at least 50 employees, where a majority of workers are exposed to difficult working conditions, in France; increasing the number of inspections focusing on the working conditions of older workers, in Portugal. A few examples addressing preventive and health promotion measures were identified, especially in northern Europe: the MASTO project, overseen by the Finnish Ministry of Social Affairs and Health from 2008 to 2011, promoted practices to increase wellness at work, such as the new centers for well-being at work, to prevent the onset of depression, provide treatment and rehabilitation to cope at work or to return to employment, and reduce cases of work disability due to depression; periodic health checks (Pago) in Netherlands; information initiatives directed to workers and managers in Germany, to make them more aware of health issues and responsible for the state of their own health.

The concept of a “culture of health” could not be identified in scientific literature or even gray literature reporting initiatives in Europe, especially involving companies and with an explicitly objective of lowering the burden of NCD and improving the health and well-being of a workforce. However, programs sponsored by global companies also based in the EU have initiated “drivers” based on the Salutogenesis theory. A report from Optum (2017), a division of United Health Group, involved a research survey of 273 multinational employers (3,000 or more employees) located in Asia-Pacific (APAC); the United Kingdom, Europe and United Arab Emirates (EMEA); or Latin America that offer two or more health and well-being programs. Findings show that the top five well-being and health programs offered in EMEA region were: relationship with near-site clinic; gym membership discounts; onsite/worksite fitness center; health risk assessments; weight management program. The bottom five well-being and health programs offered in EMEA region were: biometric health screenings; case management programs; access to onsite health specialist; health advocacy service; onsite medical clinics/worksite clinics, tobacco cessation program and flu vaccines. When questioned, about half of the employers believed that their employees' well-being was outstanding. Half of the employers surveyed believed that their company has a firmly established culture of health ownership and that culture is important or even extremely important. The private sector, particularly large corporations, has a tremendous influence on culture and is integral to achieving high social and health standards for all stakeholders, including employees. Increasingly, shareholders, investors, boards, and executives are prioritizing business values and citizenship, as well as financial measures, knowing that these affect public perception, brand, and long-term sustainability.

A growing number of companies recognize their ability to contribute to a Culture of Health and have been using their reach and influence to improve the health and well-being of employees, families, and the communities where they operate. By recognizing the importance of health and well-being across the value chain, businesses can reap the rewards with greater productivity and higher retention. Measures, metrics, and indicators play a key role in supporting corporate efforts. They promote an understanding of the concept, inform strategic thinking and planning, and provide a basis for assessing progress, gaps, and opportunities (Whitmore et al., 2018).

Novo Nordisk, known for its work in diabetes care, has expanded its focus to include a long-term, sustainable commitment to obesity treatment and prevention. The company has an industry-leading obesity pipeline in development to help those living with excess weight or obesity achieve meaningful and sustainable weight loss, but it recognizes that the bias and stigma which surround obesity today will hinder the effectiveness and adoption of any medical treatment option. To combat this stigma, Novo Nordisk is partnering with the broad community on education and advocacy, increasing access to care, and advancing medical management. Changing the social norms around obesity will ensure that patients can and do seek out treatment, and more healthcare providers offer affordable, evidence-based, medical care for obesity, both improving the lives of people living with obesity and creating a stronger market for Novo Nordisk's products.

ABInBev, the largest beer producer in the world, has supported many approaches to curbing harmful drinking over the years. This global company supports research and partnership with academics and communities to study further the population impact on such partnerships [for more details see: https://abinbevfoundation.org/leadership].

Each of these cases demonstrates the multidimensional aspects that affect well-being and health promotion, particularly as it relates to the built environment and socioeconomic factors as shown in Figure 1.

However, there are barriers that must be overcome in order to establish a successful industry/academia/community collaboration (Kilpatrick et al., 2017).

### The Relevance of Education and Training for all End Users: Professionals, Citizens, and Policymakers

Building on the Salutogenesis model, Green and Kreuter explored the complexity of health promotion education and planning in the creation of the PRECEDE-PROCEED model (Green and Kreuter, 1999). This model distinguishes five phases in the planning of health education strategies holistically, considering different levels: individuals, society and healthcare system. The model states that health education should start with a common diagnosis to determine people's perceptions of their own needs or quality of life, and their aspirations for the common good. The second phase is the epidemiological diagnosis and aims to determine which health problems are important. In the behavioral and environmental diagnosis, the third phase, the main determinants of the health problem are analyzed. In the educational and organizational diagnosis, an analysis is made of the predisposing, reinforcing and enabling factors that should be changed to initiate and sustain a process of behavioral and environmental change. These factors are the immediate targets of a health promotion program. The fifth phase is the administrative and policy diagnosis and focuses on developing health education and health regulation actions. These factors are depicted in Figure 1 which show the multifactorial and interactive antecedents to well-being.

Aging of the population in western societies and the rising cost of health and social care are refocusing health policy on health promotion and disability prevention among older people. However, efforts to identify at-risk groups of older adults and to alter the trajectory of avoidable problems associated with aging by early intervention or multidisciplinary case management have been largely unsuccessful. This failure arises from the dominance in primary care of a managerial perspective on healthcare for older people and proposes instead the adoption of a clinical paradigm based on the concept of “one health” across all policies. On these bases, professionals from the social and health domains should be trained in order to effectively and collaboratively meet the needs arising from the present socio-demographic situation.

Shortcomings have been noted in undergraduate curricula worldwide with regard to content about the multi-domain approach to health (Cano et al., 2018; Windhaber et al., 2018). The challenge for health professionals is to stimulate undergraduate interest in cross-sectoral training where the burden of an increasing prevalence of older people suffering chronic illness and multiple comorbidities can be sustained only with a paradigmatic shift toward a proactive attitude. Efforts to expand the health professional curriculum in the EU countries have begun, for example, nursing education focusing on community-based competencies for aging populations, as reported in the EnHANCE [www.enhance-fcn.eu; Nr 2017-2976_591946-EPP-1-2017-1-IT-EPPKA2- SSA- Ref.17D027253]. Evidence suggests that the management of acute illness associated with hospitalization dominate medical curricula. Managing frailty and multimorbidity mostly regard the geriatrics medical specialty, whereas to promote active and healthy aging there is a need for close collaboration and communication along the entire life-course, across specialties, and between professionals (psychologists, sociologists, communication experts, social workers) and caregivers.

### Fostering Compassion in Health Care Systems

While the aging of societies around the world, particularly in the EU (Rechel et al., 2013), is to be celebrated, it is associated with many challenges (Cano et al., 2018), the most important of which is the provision and rationing of appropriate and timely care to the growing number of older adults with frailty, a multi-factorial vulnerability to adverse outcomes associated with disability and co-morbidity (Clegg et al., 2013; Rodríguez-Mañas et al., 2013). Frailty is now recognized for the first time as an emerging public health emergency requiring urgent attention (Cesari et al., 2016). This has led the EU to prioritize policy and research funding targeting preventive strategies that promote active and healthy aging (Bousquet et al., 2014, 2017; Buckinx et al., 2015; O'Caoimh et al., 2015; Michel et al., 2016). An understanding of the epidemiology of frailty is important to develop not only appropriate responses but also effective preventive measures, ideally before the onset of functional decline (Plough, 2015). To support this, the European Commission recently funded the Joint Action on Frailty Prevention, ADVANTAGE (grant number #724099). This aims to develop a holistic and comprehensive strategic framework for the prevention and management of frailty at the European level, bringing together partners from 22 European countries. Public health plays a central role in shaping a shared Culture of Health (O'Caoimh et al., 2018). It is essential to develop a fair and equitable roadmap for frailty prevention toward active and healthy aging, and to embed a public health approach as part of Europe's Culture of Health. This encompasses a better understanding of the risks associated with developing frailty including the factors that drive frailty transitions from non-frail and pre-frail to frailty and back, some of which are socially determined (Rodríguez-Laso et al., 2018). It will also be important to help establish robust processes and systems for the screening, monitoring, and surveillance of frailty at population-level in order to intervene and prevent the onset of functional decline (Rodríguez-Laso et al., 2018). Preventing and managing the challenge of an aging population requires the strengthening of compassion into our health systems. Compassion centers on the ability to understand the emotions of others combined with a desire to assist and promote their well-being (Perez-Bret et al., 2016; Sinclair et al., 2018). Although it is an important, albeit often overlooked element contributing to our culture of health, providing compassion in busy everyday practice let alone at the policy level is often a challenge in the face of competing for demands and compassion fatigue (Fernando and Consedine, 2017). Many factors contribute to this, but the capacity to deliver compassion in healthcare at the individual level can be enhanced through organizational support and education (Zamanzadeh et al., 2018). Understanding older people and their life experience are also crucial in fostering compassion. In particular, it is necessary to appreciate that an older persons' outlook and perspective on life can impact on their perceived health, quality of life (Zamanzadeh et al., 2018) and ultimate life expectancy (Department of the Premier Cabinet, 2013). These change over time such as a river or hourglass flow over the human lifespan (Antonovsky, 1979; Strough et al., 2016). As we age, many older people perceive the life-span hourglass as being half empty such that they focus more on limited time and less on future opportunities; this affects their ability to initiate preventative strategies to improve their health (Cockburn et al., 1999).

### Health Equity Policy and Research Actions to Advance a Shared Culture of Health in EU

The right to health currently finds significant differences between the various social groups, as well as among territorial areas, both with regard to risk factors and to the real access to services and healthcare. The increasing levels of health inequalities in the EU and related growing economic discrepancies result in increased cost of individual well-being and social-health services and coagulate the main determinants of the gap in life expectancy among the different socio-economic groups. The last economic crisis exacerbated these gaps, especially for families, whose composition in Europe has progressively changed, evolving from the traditional family nucleus to forms of de-standardization of the family composition (Brückner and Mayer, 2005; Jokinen and Kuronen, 2011; Huinink and Kohli, 2014). These changes parallel the complex evolution in Europe of social contexts which influence family dynamics (Hobson and Olah, 2006; OECD, 2011). There are substantial differences among European regions in the social services for families, with a north-south gradient in social support and pronounced gender differences in social positions covered (Lewis, 2006; Saraceno, 2008; Olah, 2015), that translates in health inequalities. The recent economic crisis had implications for family stability and birth trends (Sardon, 1993; Philipov and Dorbritz, 2003; Frejka et al., 2008; Hiekel and Castro-Martin, 2014; Olah et al., 2014; Perelli-Harris et al., 2014), with an increase of extended family units in southern Europe. The increase in NCCD entails not only health service demands but also the need for the creation of a Culture of Health as a shared “social good.” Individual and collective awareness of health as a common good implicates an active role of citizens, through forms of collective participation in choices and sharing between operators and users, in order to implement programs to promote healthy lifestyles, primary disease prevention, and NCD management. There is a need for a change in the design and management of social, welfare, and healthcare models to become more integrative and synergic across domains. This can be recomposed through the construction of formal and informal collaborative networks between welfare and health that is digitally supported, to make health and social planning accessible to new development models, such as “living labs.” Living labs allow the scale-up of new and sustainable approaches to health in the context of health promotion and disease prevention, such as innovative adapted physical activity programs, cognitive training, and primary nutritional interventions.

Empowering citizens for active aging should hence be a priority in all policies. This is stated by WHO (WHO, 2002) specifically relating to actions to reduce risk factors associated with major diseases and increase factors that protect health throughout the life course: promote regular, moderate physical activity and prevent malnutrition ensuring food security and safety, while enhancing social cohesion, as people age. Evidence shows that individuals with higher education are more prone to a positive and durable lifestyle change than those with lower education, for whom achieving a positive change is more difficult (Nilsen et al., 2015). According to the Salutogenesis model, low individual or population education can translate into a significant health problem. Hence it is necessary to intervene in all the components of this model: the ability to understand what happens (cognitive), the ability to manage the situation (behavior) and the ability to find meaning in the situation (motivation) (Benz et al., 2014). It is necessary to give the individual(s) or populations the ability to use their own resources than the resources themselves in adopting a more active lifestyle. It is also necessary to educate people on healthy balanced and safe food intake, personalized upon individual needs and taste and cultural preferences (Di Furia et al., 2016; Vuolo et al., 2016; Di Somma et al., 2017).

The A3 Nutrition Group of the EIP on AHA developed an integrated view on a common nutritional approach to frailty focused on a step-wise approach to malnutrition. This approach links assessment to adequate interventions (primary/secondary/tertiary) and is aimed to implement innovative tools for effective social connectivity, prevention, detection, and treatment measures (Illario et al., 2016).

Several examples exist of such an approach, including the Mediterranean Diet, that is grounded on the sociocultural background of the emblematic communities (Moro, 2016) and has shown an extraordinary impact on health, including social connectivity (Bonaccio et al., 2018).

European policies on health promotion would benefit from the Salutogenesis model to generate more effective outcomes: for example, promoting health literacy in all populations, and knowledge of the impact of the adoption of healthy lifestyles in all end users (schools, health professionals, urban planning, transportation, sports and recreation, research, etc.). This means increasing the ability to understand what happens in the events of life, as also stated in the first strategic objective—create an active society—building the awareness that it is never too late even when you are older to start being more active under the guidance of trained professionals (health professional, physiotherapist, exercise professional, nutritionist, dietitian, endocrinologist, chef) that collaborate to outline the appropriate intervention, such as the correct level of intensity for the desirable physical activity or food uptake. Specifically, for older adults, a greater concern on avoiding musculoskeletal lesions and falls and enhance coordination and balance should be present, associated with adequate intake of vitamin D to prevent osteoporosis, and of proteins to prevent sarcopenia. In some populations, walking and manual activities are an important component of a community exercise program (Tomás et al., 2018).

The increment in technologies for supporting the empowerment of all citizens, especially older adults, to manage their active lifestyles safely, should increase in all Europe. This will enable, support, or encourage strategies that are effective in preventing or managing NCDs and in maintaining functionality sustainably. When looking for the second component of SOC on the Salutogenesis theory—the ability to manage the situation (behavior), a vast work is a need of active lifestyles and the capability of use all the resources available (WHO, 2010b).

The third component—the ability to find meaning in the situation (motivation)—is often the key to the success of an intervention aiming to decrease inactivity levels or tackle malnutrition. For example, in programs aiming to reduce overweight and adhere to adequate food intake, it is for some participants more critical to work initially on motivation and only afterward focus on the exercise and dietary changes as their social connectivity increases. When translating WHO strategic objectives, the second strategic objective—Create active environments—could also contribute to this component. In fact it states that environment should motivate to be more safely active for all ages, specifically for older adults who frequently have other comorbidities impacting movement, and therefore the built environment should be planned for their safety and pleasure, contributing to healthier lifestyles sustainability (Sallis et al., 2016).

## Conclusions and Recommendations for Future Research

As our population is aging, and NCDs are increasing across the globe, we need to set priorities that are based on the Salutogenesis model, and will not only assist individuals with NCD to strife toward a healthier status and higher level of well-being, but also contribute to creating a culture of health that promotes well-being throughout the entire lifespan (Baum et al., 2018). The following key areas should be among the top priorities in maintaining a culture of health: leadership training for change in management models; cross-disciplinary teamwork; citizens engagement as co-founders of a culture of health; personalized approach toward different age groups (children, teens, young adults, adults, older adults); private-public alliances to promote a culture of health; sharing of knowledge, skills and tools available and accessible for a digitally empowered society; and experiential education of policymakers (Stenberg et al., 2017).

Establishing programs that support co-creation leaders is a key driver for creating synergies in domains suffering from polarization, inertia and transforming problems into opportunities for innovation through peer-to-peer interactions (Ackoff, 1989). Indeed, the stakeholders' attitude toward self-empowerment and co-creation of well-being is an important variable in establishing a “culture of health.”

Utilizing the framework of the 13 domains identified by the Joint Action “CHRODIS” (Grant number #20132201); we will be able to promote change at the local level (JA-CHRODIS Project Consortium, 2015) to reduce the burden of chronic diseases (FAO, 2013). Research in this field should focus on large studies that allow translating information in policies for promoting health (Reis et al., 2016), as well as to identify predictors of disability and functional decline, and the factors that contribute to increasing adherence to physical activity, to healthy food intake and to an active lifestyle.

Another focus of research should be on interventions that actually produce results for the population they were designed for. The appropriate program for the appropriate intervention in the appropriate cultural setting is needed. The identification of good practices in physical activity promotion and dietary habits, among older adults and also in the youngest should be incremented as well as the analysis of social benefits and impact of those policies.

## Author Contributions

CT-S, VD, and MI conceived the presented idea, drafted the table of contents, and wrote all the manuscript. SS contributed to the Abstract, Background, and Creating Synergies Among all Stakeholders to Strengthen Wellbeing in the Workplace: the Contribution of Business/Companies and Health Care Facilities sections. PSc, CC, GG, and LCo contributed to Abstract, Background, and Defining the Culture of Health in the Framework of Societal Challenges sections. VZ contributed to Case Studies of Salutogenesis Model in NCD section. GL contributed to Multilevel and Multidomain Wellbeing Assessment and The Case for Older Adults sections. GI contributed to Co-development of Technological Innovations with End-Users section. CG contributed to Background section. DT, RR, and GT contributed to Background, Defining the Culture of Health in the Framework of Societal Challenges, and Health Equity Policy and Research Actions to Advance a Shared Culture of Health in EU sections. MT, BF, and SM contributed to Health Equity Policy and Research Actions to Advance a Shared Culture of Health in EU section. JA contributed to Abstract, Background, and Defining the Culture of Health in the Framework of Societal Challenges sections. AC and LL-S contributed to Fostering Compassion in Health Care Systems section. A-MP and AH contributed to Creating Synergies Among all Stakeholders to Strengthen Wellbeing in the Workplace: the Contribution of Business/Companies and Health Care Facilities section. RO and WM contributed to Abstract, Background, Defining the Culture of Health in the Framework of Societal Challenges, and Health Equity Policy and Research Actions to Advance a Shared Culture of Health in EU sections and critical reading. LCa contributed to Abstract, Background, and The Relevance of Education and Training for all End Users: Professionals, Citizens, and Policymakers sections. HJ-W contributed to Abstract and Background sections. MT, PSp, and VV contributed to Health Equity Policy and Research Actions to Advance a Shared Culture of Health in EU and Conclusions and Recommendations for Future Research sections and critical reading.

### Conflict of Interest Statement

The authors declare that the research was conducted in the absence of any commercial or financial relationships that could be construed as a potential conflict of interest.
